# Re-Analyses of Samples From Amyotrophic Lateral Sclerosis Patients and Controls Identify Many Novel Small RNAs With Diagnostic And Prognostic Potential

**DOI:** 10.1007/s12035-025-04747-2

**Published:** 2025-02-21

**Authors:** Phillipe Loher, Eric Londin, Hristelina Ilieva, Piera Pasinelli, Isidore Rigoutsos

**Affiliations:** 1 Computational Medicine Center, Thomas Jefferson University, Philadelphia, PA 19107 USA; 2 Weinberg ALS Center, Vickie and Jack Farber Institute for Neuroscience & Department of Neuroscience, Thomas Jefferson University, Philadelphia, PA 19107 USA

**Keywords:** miRNAs, isomiRs, tRNAs, tRFs, rRNAs, rRFs, Y RNAs, yRFs, Diagnostics, Prognostics, Amyotrophic lateral sclerosis, ALS, Burkholderiales, Nucletmycea

## Abstract

Amyotrophic lateral sclerosis (ALS) is a highly heterogeneous disease for which accurate diagnostic and prognostic biomarkers are needed. Toward this goal, we reanalyzed two published collections of datasets generated from the plasma and serum of ALS patients and controls. We profiled these datasets for isoforms of microRNAs (miRNAs) known as isomiRs, transfer RNA-derived fragments (tRFs), and ribosomal RNA-derived fragments (rRFs), placing all remaining reads into a group labeled “not-itrs.” We found that plasma and serum are rich in isomiRs (canonical, non-canonical, and non-templated), tRFs, rRFs, and members of an emerging class of small RNAs known as Y RNA-derived fragments (yRFs). In both analyzed collections, we found many isomiRs, tRFs, rRFs, and yRFs that are differentially abundant between patients and controls. We also performed a survival analysis that considered Riluzole treatment status, demographics (age at onset, age at enrollment, sex), and disease characteristics (ALSFRS, rD50, onset type) and found many of the differentially abundant small RNAs to be associated with survival time, with some of these associations being independent of Riluzole treatment. Unexpectedly, many not-itrs that did not map to the human genome mapped exactly to sequences from the SILVA database of ribosomal DNAs (rDNAs). Not-itrs from the plasma datasets mapped primarily to rDNAs from the order of Burkholderiales, and several of them were associated with patient survival. Not-itrs from the serum datasets also showed support for rDNA from Burkholderiales but a stronger support for rDNAs from the fungi group of the Nucletmycea taxon. The findings suggest that many previously unexplored small non-coding RNAs, including human isomiRs, tRFs, rRFs, and yRFs, could potentially serve as novel diagnostic and prognostic biomarkers for ALS.

**Supplementary Information:**

The online version contains supplementary material available at 10.1007/s12035-025-04747-2.

## Introduction

ALS is a progressive degenerative disease of motor neurons that results in paralysis of most skeletal muscles, including the diaphragm, leading to death from respiratory failure typically 3–5 years after the onset of symptoms, although disease duration varies significantly [1, 2]. In up to 50% of cases, there is also degeneration in the frontal and temporal regions of the brain, leading to cognitive dysfunction and, in 15% of the cases, to frontotemporal dementia (FTD) [3], which is characterized by abnormal behavior and speech [1, 2]. Overall, it is reported that approximately 50% of ALS patients have cognitive decline or behavioral changes like those observed in FTD [4].

While 90% of cases present as single (sporadic) cases without a family history, in the remaining 10% of cases, there is a family history. To date, as many as 40 genes have been determined to cause familial ALS. Four of the 40 genes – C9orf72, SOD1, TARDBP, FUS – are mutated in approximately 70% of patients with familial ALS [2]. Because ALS is a highly heterogeneous disease at the clinical, genetic, and pathological levels, there is an urgent need for biomarkers that can sensitively and specifically identify the disease, categorize patients based according to etiology, and improve prognosis [1].

The most studied ALS biomarkers in biofluids are the neurofilament light chain (NfL) [5, 6] and the microRNAs (miRNAs) [7]. More recently, others [7–16] and we [17–29] have been exploring additional classes of sncRNAs as candidate biomarkers in tissues and biofluids. These classes include the isoforms of miRNAs that are known as “isomiRs” [30]; the fragments of transfer RNAs (tRNAs) that are known as “tRNA-derived fragments” or tRFs [31, 32]; and the fragments of ribosomal RNAs (rRNAs) known as “rRNA-derived fragments” or rRFs [19–21]. While sncRNAs have shown promise as biomarkers for the prognosis and diagnosis of various diseases and conditions [18–20, 24, 25, 33], their exploration in the context of neurological disorders [7, 8, 14, 16, 21, 34, 35] has not been as widespread as in cancers.

Here, we present our findings from the re-analysis of two collections of datasets generated from the plasma and serum of ALS patients and controls. We systematically analyzed and compared the profiles of isomiRs, tRFs, rRFs, and of all remaining small RNAs not belonging to these three categories (“not-itrs”) in the two biofluids. Lastly, for all sncRNAs that satisfied abundance thresholds, we examined their ability to serve as novel candidates for the diagnosis and prognosis of ALS.

## Methods

### Patient and Control Datasets

From NIH’s Gene Expression Omnibus (GEO) [36], we downloaded the plasma-derived short RNA-seq datasets described in [7] with accession number GSE168714 and the serum-derived short RNA-seq datasets described in [14] with accession number GSE148097. Both collections include samples obtained from ALS patients and controls. For our non-longitudinal analysis using the plasma-derived datasets, our diseased cohort comprised the same n = 248 patients used in the combined cohort of [7]. For the longitudinal analysis we utilized the T1 and T4 time points for all 22 participating patients of that cohort. See also Supp. Table S1.

### Data Preprocessing

The plasma collection (GSE168714) used unique molecular identifiers (UMIs) [37] during library preparation. We removed UMIs from the sequenced reads using the filtering strategy described in [38]. For the purpose of UMI-deduplication, we leveraged the open-source “UMI-Tools” package [39]. We used this package’s ‘*extract*’ utility to remove the 3´ adapter and identify the UMI tag for each read, then discarded any reads that did not contain a 3´ adapter. We used ‘*count_tab*’ to form clusters with shared tags and remove duplicates (“de-duplication” step). We only kept reads with a minimum length of 15 nts. For the serum samples (GSE148097) that did not include UMIs, we used ‘*cutadapt*’ [40] to remove adapters (error rate = 12%, quality cutoff = 15). We again kept only reads with a minimum length of 15 nts.

### Mapping sncRNAs

To profile isomiRs and tRFs in the plasma and serum datasets, we used our previously published isoMiRmap [30] and MINTmap [31, 32] tools, respectively, with default settings. To profile human rRFs we used a brute-force search of the genome, as we outlined in [19], enforcing exact matching. Note that isoMiRmap can also profile non-templated isomiRs with 3´ post-transcriptional additions – by definition, these cannot map exactly to the human genome. Moreover, MINTmap can profile non-templated tRFs with the “CCA” that is post-transcriptionally added to mature tRNAs. Analogous post-transcriptional additions are not known for rRFs. Any reads that we could not categorize as isomiRs, tRFs, or rRFs we placed into a fourth class, the “not-itrs,” i.e., not isomiRs, tRFs, or rRFs. We sought not-itrs in the human genome using the Bowtie2 [41] tool. First, we used Bowtie2 by enforcing exact matching: this revealed “Group 1,” the first of three groups of sequences that make up not-itrs. Group 1 includes reads that mapped exactly to Y RNAs, as well as reads that mapped to non-annotated genomic regions. We used Bowtie2 again, allowing exactly one mismatch, i.e., one replacement, insertion, or deletion. This revealed “Group 2,” the second of the three groups of not-itrs. Group 2 contained primarily isomiRs, tRFs, rRFs, and yRFs that did not match the genome exactly. Group 3 included all remaining not-itrs.

### Searching the SILVA Database

We searched Group 3’s sequenced reads that did not map to the human genome either exactly or with one mismatch, i.e., one replacement, insertion, or deletion in the Rel 138.2 of the SILVA database [42] of rDNAs, which we downloaded on Nov 1, 2024. Rel. 138.2 was made available on July 11, 2024. Our searches focused on the non-redundant (NR) portion of the database that is provided by SILVA and contains the sequences of 510,495 small ribosomal subunits (SSU) and 95,279 large ribosomal subunits (LSU) from bacteria (509,327), eukaryota (75,067), and archaea (21,380), respectively. The corresponding SILVA files are SILVA_138.2_LSURef_NR99_tax_silva_trunc.fasta.gz and SILVA_138.2_SSURef_NR99_tax_silva_trunc.fasta.gz. Our SILVA searches were stringent and used exact matching.

### Labeling isomiRs, tRFs, rRFs, and not-itrs

To refer to the various sncRNAs, we use the “license plate” labeling scheme we initially introduced to label tRFs [43, 44]. License plates are ideal for this purpose because they are a flexible labeling scheme, create *no labeling conflicts,* require *no brokers*, and are *genome-assembly agnostic* (and, thus, time-invariant). Because of these properties, we have extended the scheme to isomiRs [30], rRFs [19], yRFs [45], and now not-itrs. License plates have also been adopted by the miRGFF3 proposed standard [46]. For backward compatibility, in the case of isomiRs, we also use the modified miRBase labels we introduced previously [28]. Each license plate is a compact representation of a nucleotide (nt) sequence that uses 32 symbols: the ten digits and the capitalized letters of the English alphabet except for A, C, G, or T. For example, the unique license plate for the 24 nt tRF GGGGGTGTAGCTCAGTGGTAGAGC is tRF-24-RK9P4P9LH9: “tRF” indicates the molecular type (tRF in this case), “24” is the length of the tRF, and “RK9P4P9LH9” is a base-32 encoding of the nt sequence GGGGGTGTAGCTCAGTGGTAGAGC. To label isomiRs, tRFs, rRFs, and yRFs, we used the prefixes “iso-,” “tRF-,” “rRF-,” and “yRF-,” respectively. For the not-itrs of Groups 1, 2, and 3 (see above) we used the prefix “na-.”

### Differential Abundance, Survival, Enrichment, and Longitudinal Analyses

Separately for each of the four RNA classes of interest, we calculated differential abundance using the DESeq2 [47] tool and identified those sncRNAs that changed between the compared settings. If a patient contributed to the longitudinal portion of the study (GSE168714 collection), we used only the first sample collected from this patient to avoid biases in the differential abundance calculations. To run DESeq2, we only considered sncRNAs with a median of 5 or more raw reads in at least one of the compared datasets. Lastly, we filtered DESeq2 results and kept only sncRNAs that satisfied the following conditions: log_2_(fold change) ≥ 0.4; False Discovery Rate (FDR) ≤ 5.0E-02; and a mean of at least 5 reads after DESeq2 normalization.

### Survival Analysis

We used Cox models [48] to perform univariate and multivariate analyses and evaluate the ability of isomiRs, tRFs, rRFs, and not-itrs to determine time-to-death (“survival time”) from enrollment and onset. We performed these analyses by accounting for treatment status (+ Riluzole, – Riluzole) or ignoring it (+ /– Riluzole) and controlling for demographic and disease characteristics. We only used the plasma datasets from ALS patients for whom we had metadata. As with the other analyses, for patients who participated in the longitudinal portion of the study, we used only their first donated sample; consequently, only 248 ALS datasets were used for the Cox models. Abundance thresholds and DESeq2 thresholds were the same as in the differential abundance analyses. For the univariate analysis, we generated Kaplan–Meier [49] curves and statistics. To this end, we used the sncRNAs’ mean abundance to split the samples into two groups, a “lower-than-mean abundance” group and a “higher-than-mean abundance” one. We also carried out multivariate analyses to control for (i) demographics (age at onset, age at enrollment, sex) and (ii) demographics + disease characteristics (rD50, ALSFRS, onset type). In both the univariate and multivariate analyses, we only kept sncRNAs whose probability of the estimated coefficient is ≤ 0.05, and either the “likelihood ratio test” or “Wald test” resulted in a p-value ≤ 0.05.

### Enrichment Analysis – Controls Versus Patients

We performed enrichment analysis for each class of interest by comparing healthy controls and ALS patients. As with our other analyses, for patients who participated in the longitudinal portion of the study, we used only their first donated sample. For this analysis only, we lowered our abundance threshold and considered as “present” any sncRNA that had an average of at least one read (unnormalized) across all compared samples. For those sncRNAs that satisfied this constraint, we performed a two-sided Fisher's exact test between control and patient samples, seeking differences in the number of samples in each group where the sncRNA is absent or present. We adjusted all p-values using Benjamini-Hochberg (BH) correction and only kept sncRNAs whose FDR ≤ 1.0 E-09.

### Analysis of Longitudinal Data

For this analysis, we used the longitudinal datasets described in [7], where blood (plasma) samples were obtained from 22 ALS patients at four time points over 30 months. We used a two-sided Fisher’s exact test to calculate the odds ratio and p-values. A sncRNA “increased” (resp., “decreased”) from time-point T1 to time-point T4 if it increased (resp., “decreased”) in T4 compared to T1 in most of the considered samples. We computed enrichments separately for sncRNAs that were found to be significantly differentially expressed (Supp. Table S5A) and those that were predictive of survival (Supp. Table S5B). To compare differences across classes, we calculated enrichments using all classes together, and also separately per class.

## Results

**Fig. 1:**
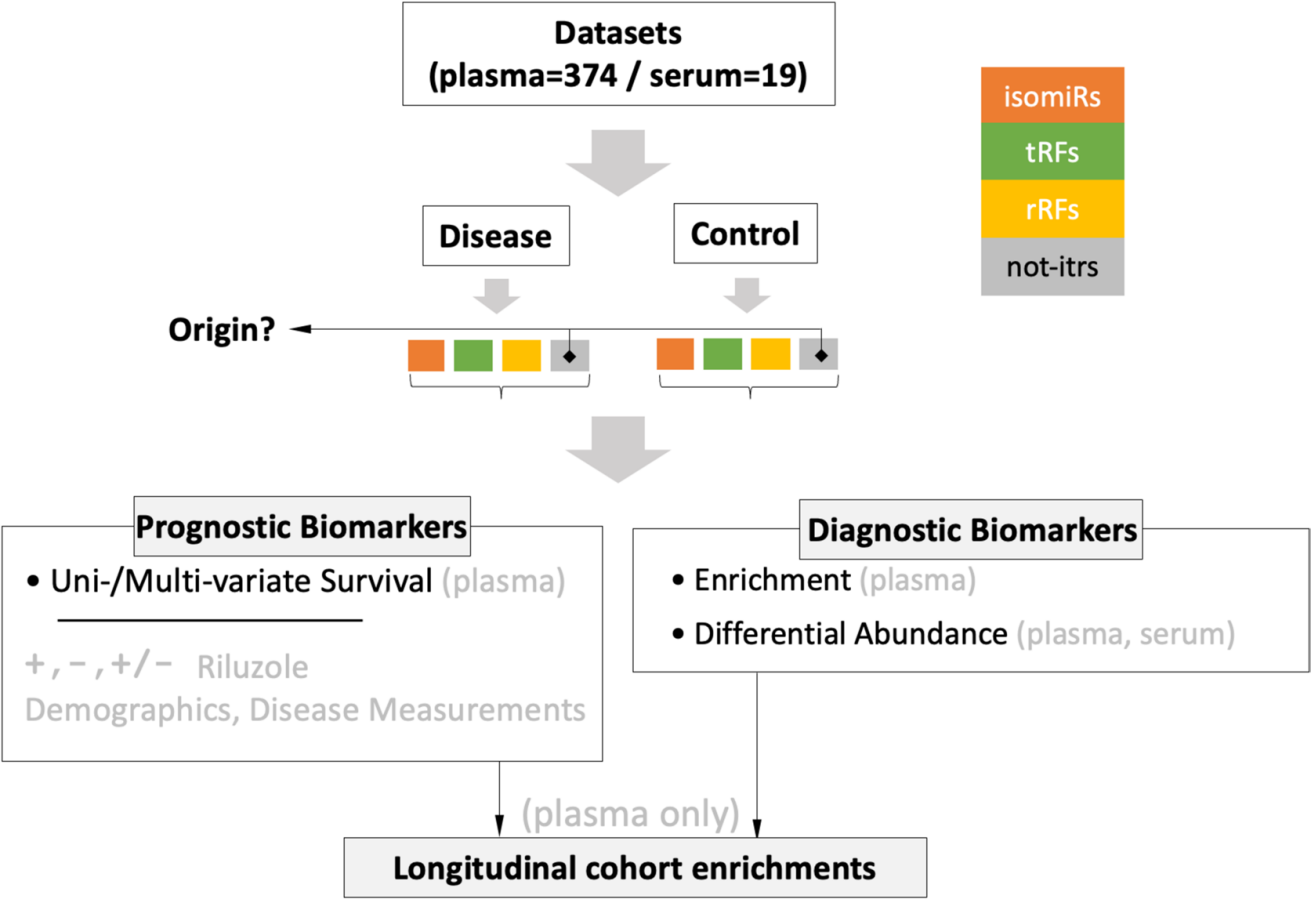
Overview of the re-analysis. We studied four classes of sncRNAs (isomiRs, tRFs, rRFs, and not-itrs) and sought to identify candidate diagnosis and prognosis biomarkers. After preprocessing the raw reads, we examined their differential abundance, enrichment, correlation, and association with survival time. We carried out the survival analysis only on the plasma collection because it included more datasets

We re-analyzed 374 plasma [7] and 19 serum [14] short RNA-seq datasets, from ALS patients and controls from GEO projects GSE168714 and GSE148097, respectively. Our goal was to investigate the presence of novel classes of sncRNAs in two different biofluids, determine the similarities and differences of the biofluids’ sncRNA contents, and explore the novel sncRNAs’ potential as diagnostic and prognostic biomarkers. Supp. Table S1 lists the datasets in each collection, their GEO and Sequence Read Archive (SRA) identifiers, and associated information. Figure 1 outlines the steps of our analysis.

### Each Dataset Contains Many Abundant isomiRs, tRFs, rRFs, and not-itrs

**Fig. 2:**
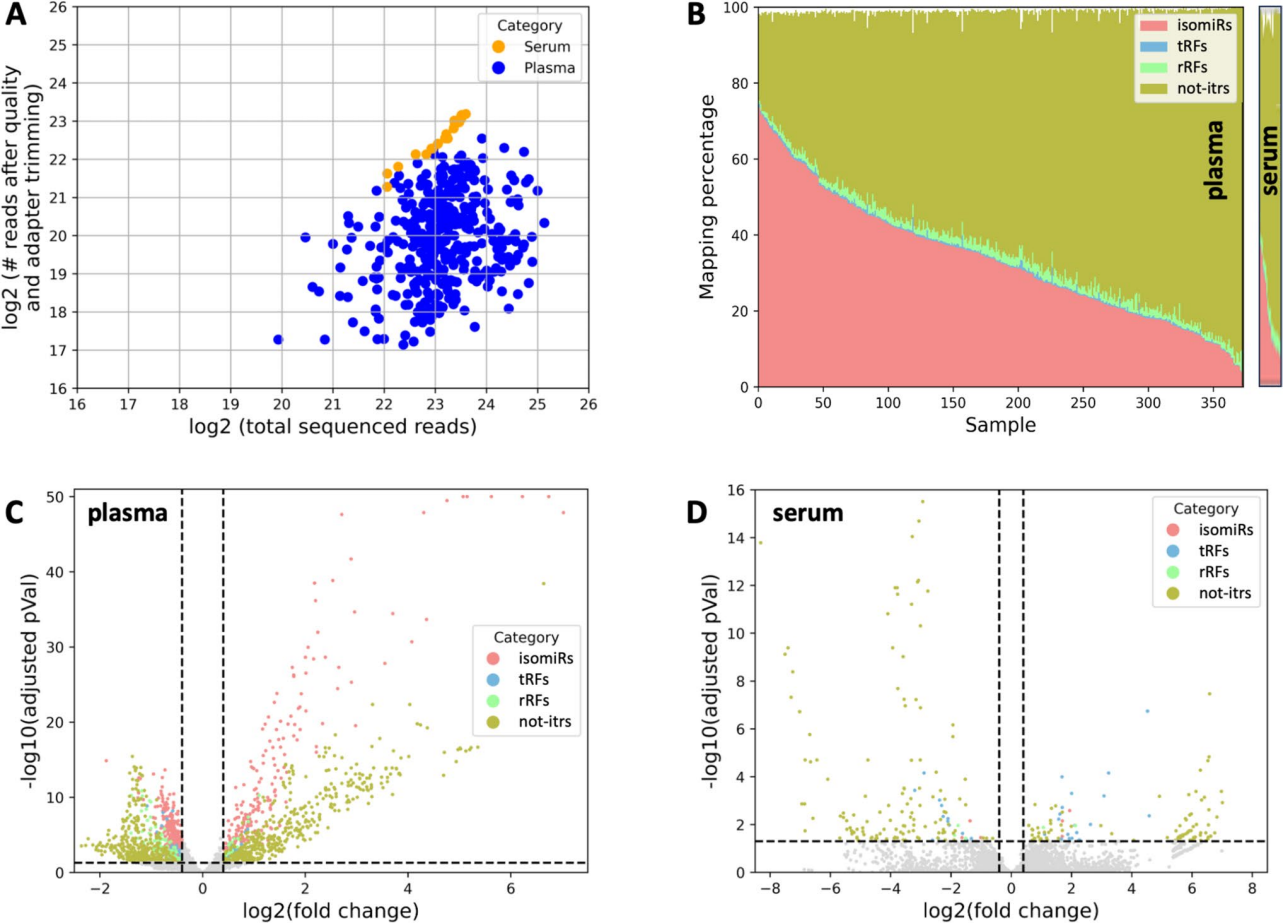
Summaries. A: Sequenced reads vs. reads that survive quality trimming and adapter removal. B: Relative abundance of the four sncRNA classes across samples: percentages do not reach 100 because only sncRNAs that are unambiguously isomiRs or tRFs are used. C-D: Volcano plots for plasma (C) and serum (D) showing which sncRNAs differ statistically significantly between patients and controls

We used publicly available tools and established approaches to quality-trim sequenced reads and remove adapters (see Methods). Figure 2A shows what fraction of the sequenced reads survive this step. Supp. Table S2 lists additional information, including the number of raw and quality/adapter-trimmed reads and the number of sncRNAs in each sncRNA class we identified in each dataset. If we consider only molecules whose abundance is ≥ 10 reads per million (RPM), the average abundance of isomiRs, tRFs, rRFs, and not-itrs across the plasma datasets is 905, 110, 221, and 2475 RPM, respectively. Across the serum datasets, the corresponding numbers are 180, 37, 574, and 7581 RPM, respectively. In other words, not-itrs have the highest average abundance in plasma and serum. Figure 2B captures the relative contributions to the sequenced reads from each sncRNA class.

Common to the plasma and serum samples is an anticorrelation in the abundance of the isomiRs and not-itrs (Fig. 2B and Supp. Table S2). For example, in the plasma dataset SRR14631778, 76.0% of all reads are unambiguous isomiRs, and 20.6% are not-itrs. On the other hand, in the plasma dataset SRR13934433, only 0.8% of all reads are unambiguous isomiRs, and 96.0% are not-itrs. The similarities between the plasma and serum collections in terms of isomiRs and not-itrs do not suggest a technical problem but rather a biological cause that is not currently understood.

### Many sncRNAs Are Differentially Abundant Between Patients and Controls

Because of the evident dichotomy in the sncRNA profiles of the samples (Fig. 2B), all our analyses examined separately isomiRs, tRFs, rRFs, and not-itrs. First, we re-analyzed the plasma samples of GEO project GSE168714 [7]. Separately for each sncRNA class, we compared the plasma samples of controls (103 datasets) with those of patients (248 datasets) using DESeq2 (see Methods). When we did not consider whether the patients were on Riluzole when the sample was collected/recorded, we found 1602 differentially abundant sncRNAs. They included 528 isomiRs, 56 tRFs, 134 rRFs, and 884 not-itrs (see Supp. Table S3A). We used DESeq2 to also re-analyze the serum samples of GEO project GSE148097 [14], which include 6 control and 13 patient datasets, and found 252 differentially abundant sncRNAs: 6 isomiRs, 34 tRFs, 5 rRFs, and 207 not-itrs (see Supp. Table S3B). Figures 2C-D show volcano plots for these differentially abundant sncRNAs.

**Fig. 3:**
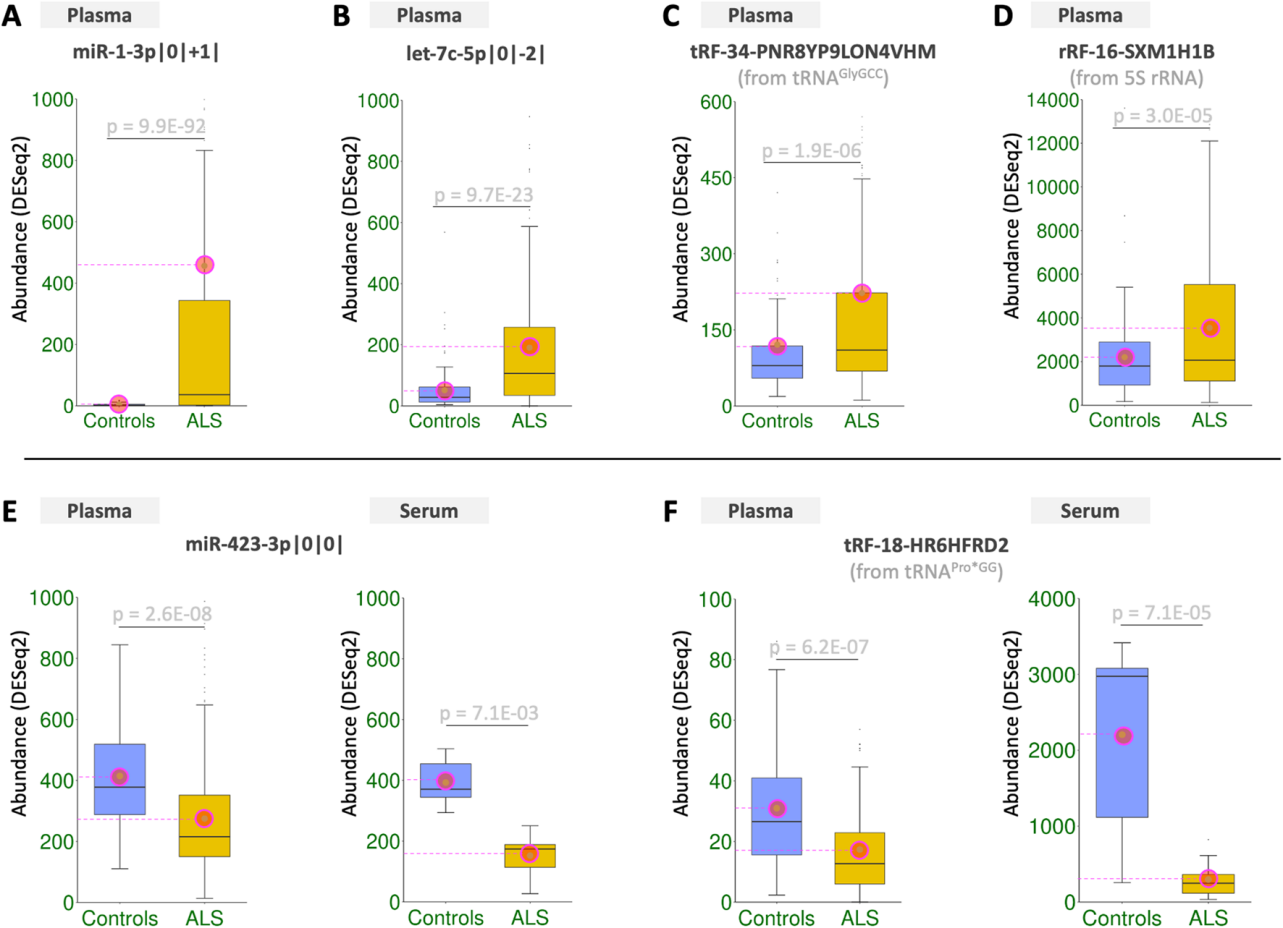
Candidate diagnostic sncRNAs in plasma and serum. A-D: Examples of several sncRNAs (two isomiRs, one tRF, and one rRF) whose abundance increases in ALS versus controls in the plasma datasets. E–F: An isomiR (miR-423-3p|0|0|) and a 3´-tRF (tRF-18-HR6HFRD2) whose abundance decreases in ALS, in both serum and plasma. The shown p-values are FDR-adjusted (as reported by DESeq2). All panels: encircled green dots mark mean values. Shown abundance levels are after DESeq2 normalization

Figures 3A-D show examples of two isomiRs, one tRF and one rRF, that are differentially abundant in the plasma datasets. Note how isomiRs miR-1-3p|0|+ 1| and let-7c-5p|0|−2| increase in abundance in ALS. The notation |0|+ 1| indicates that the miR-1-3p isomiR’s 3´ end is longer by one nucleotide compared to the reference sequence found in miRBase [28]. Analogously, |0|−2| indicates that the let-7c-5p isomiR’s 3´ end is shorter by two nts compared to the reference. The shown 5´-tRNA half from tRNA^GlyGCC^, https://cm.jefferson.edu/MINTbase/InputController?g=GRCh37&fs=GCATTGGTGGTTCAGTGGTAGAATTCTCGCCTGC&v=s&e=1.0. tRF-34-PNR8YP9LON4VHM, and the rRF https://cm.jefferson.edu/MINRbase/. rRF-16-SXM1H1B from 5S rRNA also increase in abundance in ALS. It is important to stress that examining the data at the level of isomiRs (and not the miRNA arm) improves granularity and provides invaluable resolution power. This is because not all expressed isomiRs from a given miRNA arm need to be differentially abundant or even change abundance in the same direction. If one were to consider all isomiRs from a miRNA arm together by summing up their abundances, one would risk missing consequential abundance differences between patients and controls [26]. For example, of the 30 isomiRs of miR-486-5p, the abundance of some increases in the ALS patients compared to controls, whereas the abundance of others decreases – moreover, only 10 of the 30 isomiRs from this miRNA arm change statistically significantly.

Of the sncRNAs that are differentially abundant in the plasma samples, 28 are differentially abundant in the serum samples as well, and with the same sign of change. Figures 3E-F show two such examples, the isomiR miR-423-3p|0|0|, and the 3´-tRF https://cm.jefferson.edu/MINTbase/InputController?v=s&g=GRCh37&e=1&search=submit&t=All&am=All&an=All&da=&tn=&fs=&fn=TRF-18-HR6HFRD2&c=All&st=All&gs=&ge=1. tRF-18-HR6HFRD2 from tRNA^Pro*GG^. Both molecules decrease in ALS compared to controls in both biofluids.

The plasma collection contained more datasets, which allowed us to perform Fisher exact tests (see Methods). In these tests, we sought sncRNAs enriched in either the ALS or the control group. By applying a very stringent FDR threshold of 1.0 E-09, we identified 364 sncRNAs – 58 isomiRs, 57 tRFs, 88 rRFs, and 161 not-itrs – that are enriched in either the ALS or the control group (Supp. Table S3C).

### Many sncRNAs Are Associated With Survival Time from Onset/Enrollment

To understand whether the abundance of any sncRNAs can be used to predict ALS survival (i.e., time-to-death), we analyzed the larger plasma collection using Cox proportional hazards regression and Kaplan–Meier (K-M) estimators. Our variables included Riluzole treatment status, demographics (age at onset, age at enrollment, sex), and disease characteristics (ALSFRS, rD50, onset type). Importantly, our multivariate analysis considered sncRNA abundance as a statistically significant covariate.

**Fig. 4:**
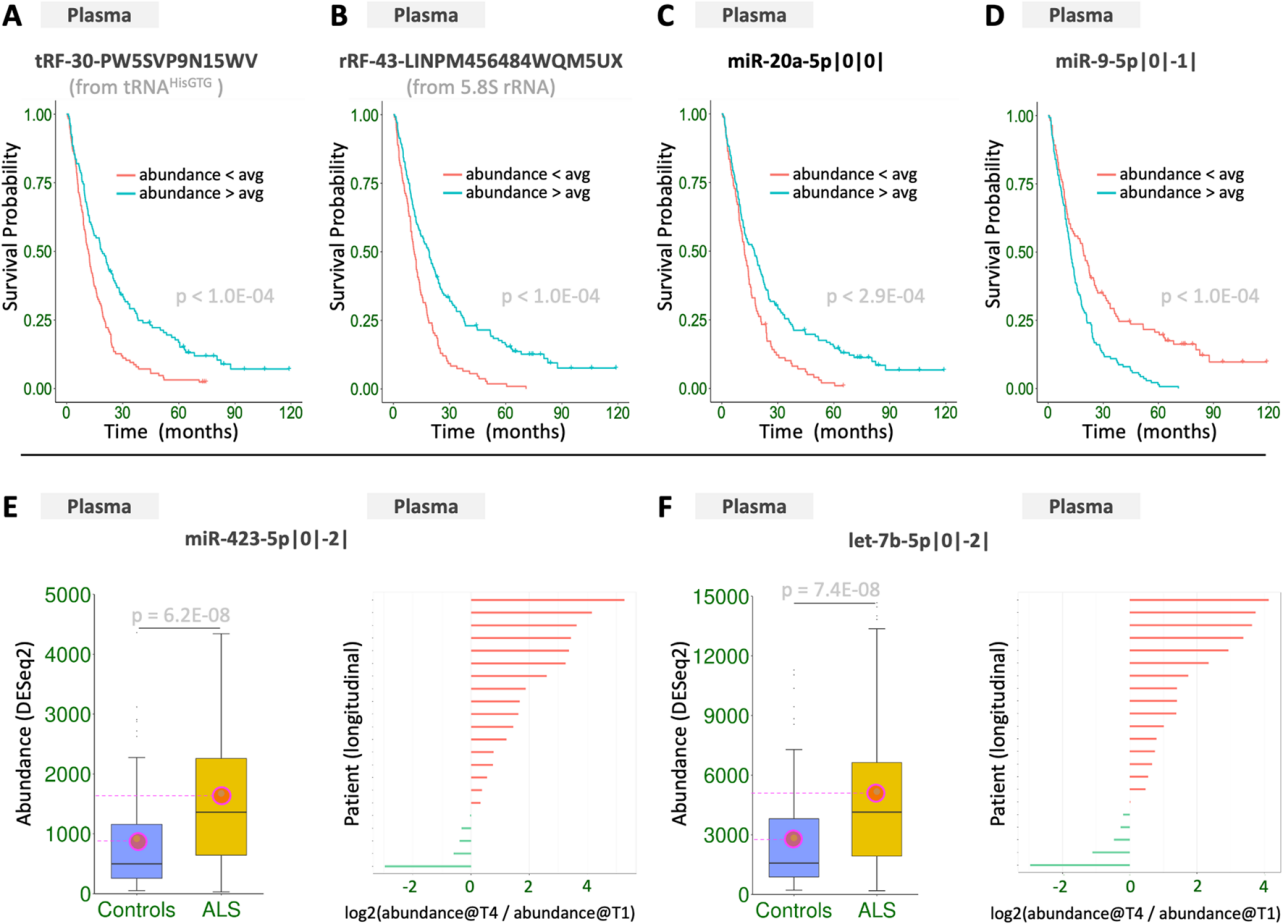
Examples of prognostic and diagnostic sncRNAs. The top row highlights a tRF (A), an rRF (B), a canonical isomiR (C), and a non-canonical isomiR (D). The bottom row highlights two non-canonical isomiRs (E–F) that have higher abundance at time T4 compared to T1 in most of the longitudinal samples (17 of 22, or 77%). The shown p-values are FDR-adjusted. Panels E–F: encircled green dots mark mean values. Shown abundance levels are after DESeq2 normalization

We identified 2701 sncRNAs that are statistically significantly associated with survival (Supp. Table S4A-C): 1020 isomiRs, 124 tRFs, 282 rRFs, and 1275 not-itrs. Three of the 2701 sncRNAs have two labels because they are listed as *both* isomiRs and tRFs in public databases, leaving us with 2698 unique sncRNA sequences. 2104 (78%) of the 2698 sequences are statistically significantly associated with survival time, whether we measure time-to-death from enrollment or disease onset. Figures 4A-D show K-M curves for four of the sncRNAs that are linked to patient survival: a 5´-tRF from tRNA^HisGTG^ (tRF-30-PW5SVP9N15WV); an rRF from 5.8S rRNA https://cm.jefferson.edu/MINTbase/InputController?v=s&g=GRCh37&e=1&search=submit&t=All&am=All&an=All&da=&tn=&fs=&fn=tRF-30-PW5SVP9N15WV&c=All&st=All&gs=&ge=. (rRF-43-LINPM456484WQM5UX); the canonical isomiR of miR-20a (miR-20a-5p|0|0|); and a non-canonical isomiR of miR-9 (miR-9-5p|0|−1|). Of the 2698 unique sncRNAs, 1977 (73%) are significant in both the univariate and multivariate analysis.

We also determined all survival associations by alternately considering and ignoring Riluzole treatment status (Supp. Table S4A-C): 2281 (84.5%) of the 2698 sncRNAs are statistically significantly associated with survival independently of Riluzole treatment status. When treatment status is considered, 1714 sncRNAs are linked to survival in untreated and 2029 in treated patients.

We note that the multivariate analysis shows age to be significant in most cases (Supp. Table S4B-C). Lastly, as expected, we found rD50 also to be significantly associated with survival (Supp. Table S4C).

### Many Prognosis-/Diagnosis-associated sncRNAs Change in Abundance as the Disease Progresses

We also re-analyzed longitudinal plasma samples from 22 patients in the GSE168714 collection. For each of these 22 patients, blood samples were collected at four consecutive time points (T1, T2, T3, T4) over 30 months. We examined samples from time points T1 and T4 to determine if any sncRNA changed in abundance as the disease progressed.

First, we compared the sign of differential change between controls and patients with the change in the longitudinal data from the subset of the 22 patients (Supp. Table S5A). We found that sncRNAs whose abundance in plasma differs between controls and patients are also more likely to change between T1 and T4 and with the same sign of change (OR = 2.11, p-value = 1.69E-09). In other words, sncRNAs with higher abundance in the ALS samples compared to controls are also more likely to have a higher abundance at time point T4 compared to T1 and *vice versa*, suggesting an underlying monotonic process. The odds ratios are statistically significant for isomiRs (OR = 4.50, p-value = 5.03E-14), and rRFs (OR = 43.36, p-value = 2.79E-11). Notably, the odds ratio for rRFs is unexpectedly very high. Figures 4E-F show two isomiRs, miR-423-5p|0|−2| and let-7b-5p|0|−2|, whose abundance increases in 17 of the 22 longitudinal samples.

We also found that sncRNAs that are predictive of time-to-death using Cox models are more likely (OR = 1.51, p-value = 6.20E-06) to change between T1 and T4 (Supp. Table S5B). Here, the odds ratios are statistically significant for survival-predicting isomiRs (OR = 2.21, p-value = 2.49E-08), tRFs (OR = 4.31, p-value = 1.86E-03), rRFs (OR = 3.95, p-value = 9.04E-06), and not-itrs (OR = 0.72 p-value = 2.30E-02).

### Many not-itrs That Change With the Disease Arise From Y RNAs or Have isomiR, tRF, or rRF Counterparts

Supp. Table S6A contains 1677 not-itrs statistically significant in at least one discussed comparison. Many of these 1677 not-itrs are abundant as shown in Supp. Table S6B (plasma datasets) and Supp. Table S6C (serum datasets). We found that by allowing one mismatch, we could map 780 of them to regions of the genome that are annotated as miRNAs, tRNAs, or rRNAs. 707 not-itrs match isomiRs with a single change (column O of Supp. Table S6A), 32 match tRFs (column P), and 43 match rRFs (column Q). Two of the not-itrs, na-19-HR0VX61I and na-20-R29P4PI3, match both an isomiR and a tRF and, thus, counted twice.

We also explored the not-itrs of Supp. Table S6A that could not be mapped to isomiRs, tRFs, or rRFs for possible instances of yRFs, an emerging category of sncRNAs [50]. To carry out this search, we assembled a list of all complete and partial human Y RNA sequences (Supp. Table S6D): 139 not-itrs mapped exactly to Y RNA sequences from this list. These not-itrs are indicated in column S of Supp. Table S6A. An additional 153 not-itrs mapped to Y RNA sequences only after allowing one replacement, insertion or deletion, and are indicated in column T. Of the 292 not-itrs that map to Y RNAs, exactly or after allowing a single change, we found that 230 arise from the 5´ and 3´ regions of RNY4. Supp. Table S6E shows these not-itrs aligned to the full-length RNY4.

**Fig. 5:**
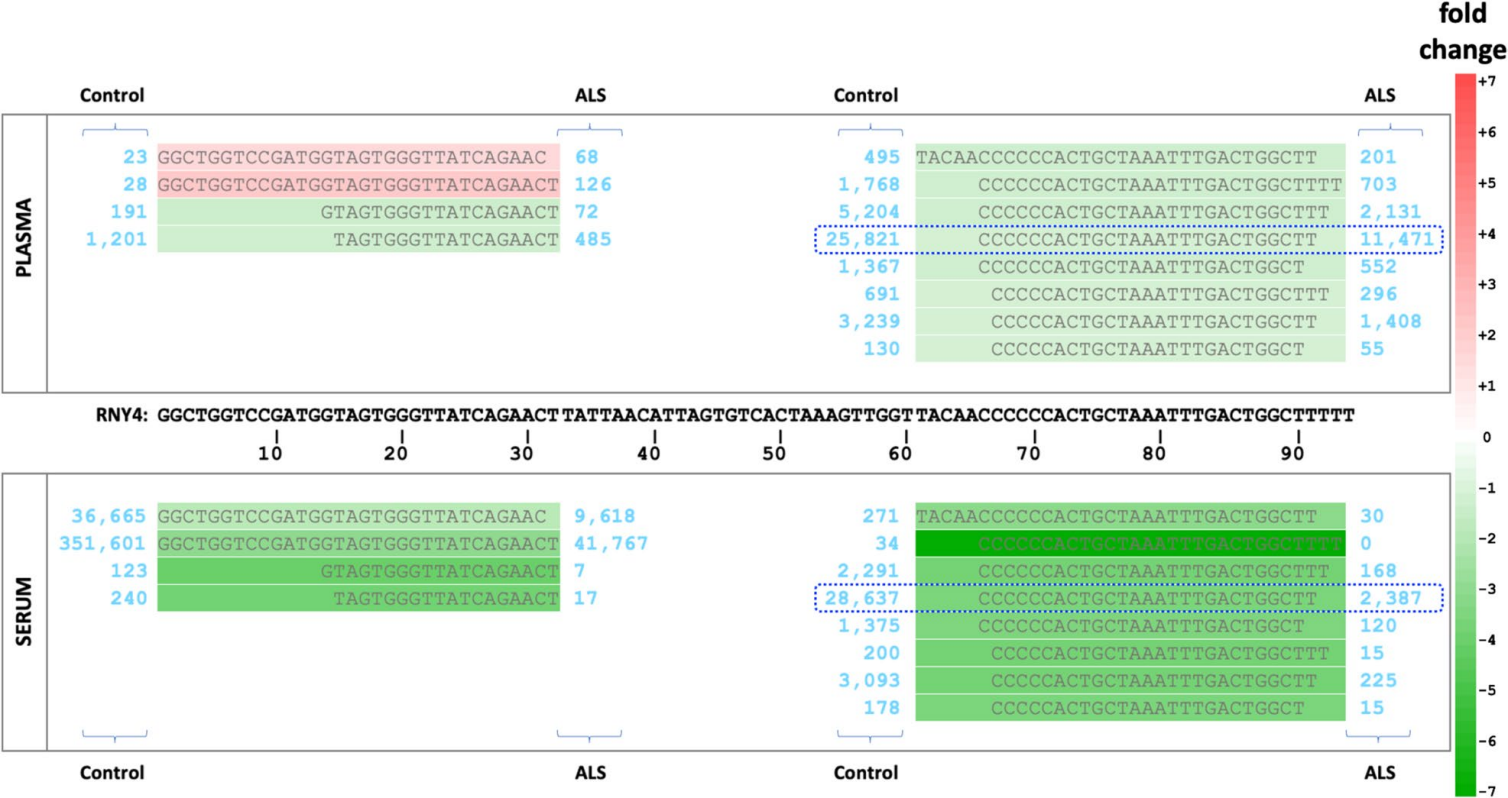
SncRNAs from RNY4. Examples of sncRNAs that arise from RNY4 and are significantly differentially abundant between ALS and controls in both plasma and serum. Only sncRNAs with a mean normalized expression of >= 50 are shown and aligned to the full-length RNY4. Shown abundance represents mean values following DESeq2 normalization

**Fig. 6:**
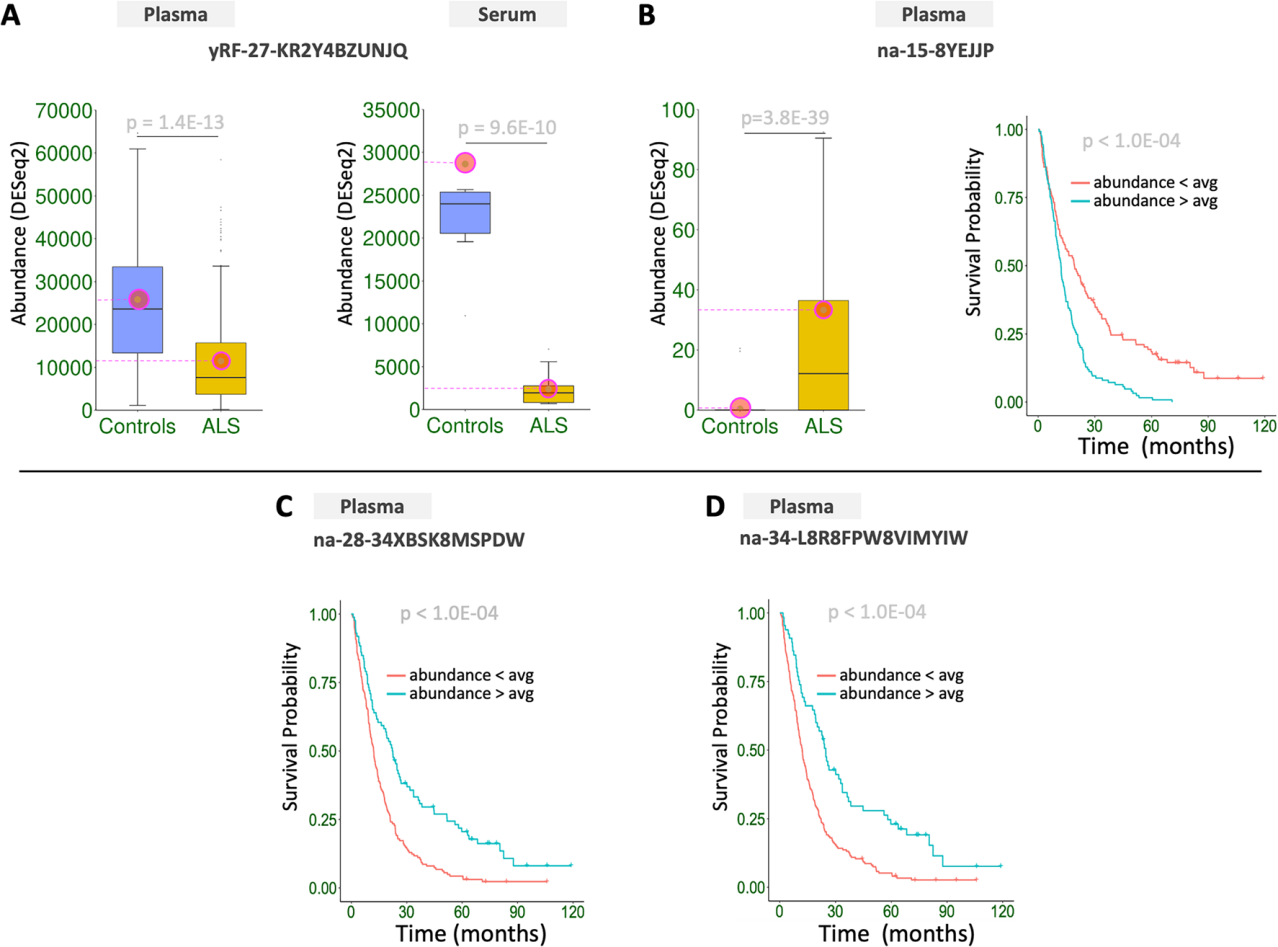
Unusual sncRNAs with diagnostic/prognostic associations. A: A sncRNA from the 3´ region of RNY4 that decreases in ALS, in plasma and serum (highlighted in Fig. 5). B: A very short sncRNA from an intron of the HOXA10 gene. C: An sncRNA that cannot be mapped to the human genome with a single change (replacement, insertion, or deletion) but maps exactly to a 5S rRNA sequence that is present in multiple bacteria. D: An sncRNA that cannot be mapped to the human genome with a single change (replacement, insertion, or deletion) but maps exactly to a tRNA-Met sequence that is present in multiple bacteria. All shown p-values are FDR-adjusted. Panels A-B: encircled green dots mark mean values. Note that in panel B the outliers are not shown because they fall outside the shown range of the Y-axis. Shown abundance levels are after DESeq2 normalization

Figure 5 shows a few characteristic yRFs that map to RNY4 exactly and whose abundance differs significantly between patients and controls in plasma and serum. Note how yRFs that differ by only 1–2 nts from one another have distinctly different abundances. Also, note the two yRFs from the 5´ region of RNY4 that are virtually absent in plasma but abundant in serum (patients and controls). Figure 6A shows boxplots for yRF-27-KR2Y4BZUNJQ: its abundance decreases in both plasma and serum in ALS patients. This sncRNA arises from the 3´ region of RNY4 and is highlighted in Fig. 5 (blue dotted rectangle). Figure 6B shows the diagnostic and prognostic power of na-15-8YEJJP, a not-itr from Supp. Table S6A that maps to an unannotated region of the human genome. This not-itr is highly enriched in ALS patients (odds ratio of 61.8 – Supp. Table S3C). In plasma, na-15-8YEJJP has a higher abundance in ALS patients. Notably, *increases* in its abundance are associated with *decreased* survival time (Supp. Tables S3A, S4). na-15-8YEJJP has a unique distinction: even though it is short (15 nts), it appears exactly once in the human genome, in an intron of the HOXA10 gene, even though 5.8 instances are expected by chance. No other annotation is currently available for this region.

Lastly, Figs. 6C-D show survival curves for two not-itrs from Supp. Table S6A that are statistically significant and cannot be mapped to the human genome, even when allowing one mismatch: na-28-34XBSK8MSPDW and na-34-L8R8FPW8VIMYIW. We examined the potential origin of these sequences through a search that used the BLAST [51] implementation at the National Center for Biotechnology Information (NCBI) of the National Institutes of Health (NIH) and targeted the “Standard databases (nr, etc.)”. The search returned concordant results for both queries: na-28-34XBSK8MSPDW matched exactly the 5S rRNA of multiple bacteria, while na-34-L8R8FPW8VIMYIW matched the tRNA-Met of multiple bacteria.

### Many not-itrs That Do ot Map on the Human Genome Likely Arise From Non-human Sources

We hypothesized that many more of the sequenced not-itrs may originate in microbes. To ensure generality, we expanded our search by including *all* not-itrs from the plasma and serum collections that did not map to the human genome assembly GRCh38 even after allowing a single replacement, insertion, or deletion. To ensure minimum abundance and that these not-itrs recur across samples, we focused on not-itrs supported by ≥ 10 reads in ≥ 20% of each collection’s datasets (76 datasets for the plasma collection and 4 for the serum collection). There are 2213 not-itrs from the plasma and 44,248 sncRNAs from the serum collection that satisfy these constraints.

We sought these sncRNAs separately among the SSU and LSU sequences of the SILVA database (see Methods) while enforcing exact matching. We ranked the SSU and LSU separately and in decreasing order of the number of distinct not-itrs found in each. We combined consecutively ranked organisms whose SILVA annotation agreed on the first five taxonomic levels (typically domain, phylum, class, order, and family for the well-annotated entries) into a single group. We labeled the organism receiving the most hits among each group’s members as the group’s representative. By definition, the support of any organism in a given group is higher than the support of all organisms in all lower-ranking groups. Supp. Tables S7  A and S7B show statistics for the top 10 groups in the four possible combinations {plasma, serum} x {LSU, SSU}. For each group, we list the number of the distinct organisms that comprise it, the labels of the corresponding five taxonomic levels, and the number of not-itrs that match exactly the sequence, SSU or LSU, of the group’s representative. As can be seen, we found exact hits in SSU and LSU sequences from numerous different bacteria and eukaryota. Supp. Tables S7A-B also make the following dichotomy apparent. Most of the plasma not-itrs that match exactly SILVA entries are of *bacterial* origin, primarily from the Comamonadaceae family in the order of Burkholderiales. The serum not-itrs also included Comamonadaceae family entries among the highest-ranking hits but most of the entries were of *eukaryotic* origin, primarily fungi from the Nucletmycea taxon.

## Discussion

We analyzed plasma and serum datasets from ALS patients and controls (Supp. Table S1), casting a wide net that comprehensively investigated novel classes of sncRNAs (Fig. 1). The classes included isomiRs (i.e., miRNA isoforms), tRFs, rRFs, and not-itrs. We found plasma and serum to be very rich in sncRNAs, with the relative proportions of each class varying from sample to sample (Fig. 2 and Supp. Table S2). Many of these sncRNAs exhibited statistically significant abundance changes between patients and controls in the two biofluids, showing their potential as candidate diagnostic and prognostic biomarkers (Supp. Tables S3, and S4).

Previous related work profiled miRNAs in plasma, serum, and cerebrospinal fluid samples from patients and controls [7, 8, 11–14, 16, 34, 52]. However, these earlier studies did not examine miRNA isoforms, i.e., isomiRs. IsomiRs capture more information than the reference miRNAs found in public databases, and their abundance levels and regulatory control of mRNAs depend on context (tissue type, tissue state, disease) and personal attributes (sex, ancestry) [19–25, 33]. Our analyses revealed 588 isomiRs whose abundance changes significantly between ALS and controls in either plasma or serum (Figs. 3–4 and Supp. Tables S3A-C). These isomiRs arise from only 157 miRNA arms from the miRNAs listed in miRBase.

We also distinguished among isomiRs that are canonical (i.e., the reference miRNAs found in miRBase [53]), non-canonical (i.e., matching the precursor miRNA sequence but differing from the reference) [33], and non-templated (i.e., not matching the precursor miRNA sequence because of post-transcriptional additions) [30, 54]. Surprisingly, we found that 241of the isomiRs that change in ALS are non-templated, whereas an additional 19 contain polymorphisms (Supp. Tables S3A-C). On a related note, the commercially available quantification assays cannot measure isomiRs correctly because of crosstalk among the co-existing isoforms [55]. This complication may be responsible for the previously reported lack of concordance between RT-qPCR and deep sequencing of biofluids from ALS patients [16, 52].

Our findings demonstrate that an isomiR-based approach provides more resolving power. To highlight this point, we mention three examples from mirna loci mir-181a, mir-1, and mir-423, respectively:

- In the case of mir-181a, the canonical miRNA from its 5p arm is known to be diagnostic for ALS [7]. Our analysis corroborated this finding while also uncovering three *non-canonical* and one *non-templated* isomiR from the same arm that decreased significantly in the plasma of patients (FDR < 1.3E-04). See Supp. Table S3.
- In the case of mir-1, our analysis identified eight isomiRs from its arm that are abundant in controls and increase three to seven times in the plasma of patients. These changes are significant (FDR < 2.1E-31) for all eight isomiRs. Three of the eight are *non-canonical*, whereas four are *non-templated* (Supp. Table S3). We note that changes in the circulating levels of miR-1 have been linked to cardiomyopathy [56], a condition previously reported in patients with motor neuron diseases [57].
- The third example pertains to two isomiRs from the mir-423 locus. The isomiRs arise from its 5p (miR-423-5p|0|−2|) and 3p arm (miR-423-3p|0|0|), respectively. Figures 3E and 4E show boxplots of their abundances in ALS and controls. This example highlights a notable instance of “arm switching:” the abundance of the non-canonical isomiR from the 5p arm *increases* in ALS, whereas that of the canonical isomiR from the 3p arm *decreases*.

We also observed abundance differences between patients and controls for many tRFs (Figs. 3–4 and Supp. Table S3). The tRFs include 5´‑tRFs and 3´‑tRFs and a few 5´-tRNA halves [58]. The link between ALS and tRNA halves is known and involves Angiogenin, an enzyme that cleaves tRNAs into halves to promote stress-induced translational repression [59]. Angiogenin is frequently mutated in ALS and Parkinson's disease [60], which would suggest that the abundance of 5´-tRNA halves decreases uniformly in ALS. However, this is not what we observe. In fact, our findings agree with a previous report that only some tRNA halves depend on Angiogenin [61]. Several differentially abundant tRFs arise from mitochondrial tRNAs, which is in agreement with the known dysfunction of the mitochondrion in ALS [62].

Members of the third sncRNA class we examined, the rRFs, also change their abundance between patients and controls (Figs. 3–4 and Supp. Table S3), with 217 rRFs decreasing in abundance in ALS. Most notable among them is an rRF from the 5´ end of 5S rRNA whose abundance increases in ALS (Fig. 3D). This concurs with the recent finding that rRF abundance depends on context and personal attributes [19, 63].

We found that many sncRNAs, including isomiRs, tRFs, and rRFs, are linked to patient survival (Fig. 4), a result arrived at through both univariate and multivariate analyses (Supp. Table S4). Moreover, analysis of the longitudinally obtained plasma samples showed that many of these prognosis-associated sncRNAs and many diagnosis-associated ones change in abundance as the disease progresses (Supp. Table S5). The sign of the observed changes suggests an underlying monotonic process.

The group of not-itrs proved to be intriguing. It contains sncRNAs that can be mapped to the human genome – specifically, miRNA precursors, tRNAs, and rRNAs – if we allow one mismatch, i.e., one replacement, insertion, or deletion (Supp. Table S6A). The group also contains sncRNAs that map exactly to full and partial human Y RNA sequences (Supp. Tables S6A columns S-T, S6D). Independent of their source’s identity, not-itrs are abundant across the plasma and serum datasets (Supp. Tables S6B-C). Regarding yRFs, most arise from the 5´ and 3´ regions of RNY4 (Supp. Table S6E), with two being very abundant in the serum but not the plasma datasets (Fig. 5). yRFs represent an untapped opportunity: while their presence in circulation is well documented [64], and their profiles are known to associate with cancer [65] and infections [45], their roles as biomarkers of neurological disorders remain largely uncharacterized [66].

Lastly, the group of not-itrs contains sncRNAs that cannot be mapped to the human genome when allowing one mismatch, with several of these not-itrs being prognostic of survival (Supp. Tables S4 and S6). Figures 6C-D show two such not-itrs; they map exactly to the 5S rRNA and tRNA-Met, respectively, of numerous bacteria, according to database searches. This finding prompted us to investigate the possible origin of all not-itrs that cannot be mapped to the human genome through searches of the SILVA database [42]. SILVA contains LSU and SSU sequences from more than 600,000 bacteria, eukaryota, and archaea. Our searches of SILVA showed that many of the not-itrs among those that cannot be mapped to the human genome match exactly *non-redundant* LSU and SSU sequences from thousands of organisms that belong to the same taxonomic divisions. The not-itrs from the plasma datasets map overwhelmingly to SILVA entries from bacteria belonging to the order Burkholderiales. The not-itrs from the serum datasets also map to bacteria from the order of Burkholderiales but most of the SILVA hits in this case are to eukaryota belonging to the taxon of Nucletmycea, which includes fungi, within the Opisthokonta clade [67].

Our study supports previously reported associations between the microbiome and ALS. In [68] and [69], the authors examined CNS tissue samples from ALS patients and controls and were able to amplify DNA from bacteria, specifically Burkholderiales, and fungi (see above and Supp. Table S7A-B). These studies, which identified rDNA in tissues, are concordant with our finding in circulation rRNA fragments from similar sources. Outside the central nervous system, there is also increasing evidence that the gut microbiome may contribute to the risk for ALS [70] and other neurological disorders [71, 72]. In fact, three studies that examined the microbiome profile in stool samples of ALS patients and controls found statistically significant differences in the bacterial profile of the two groups [73–75]. One of the two studies examined patients and controls longitudinally and found additionally that the microbiome profile changed as the disease progressed [73]. However, despite the strength of the uncovered associations, it remains unclear whether the identified abundance changes in the not-itrs of bacterial and fungal origin are causal for ALS. At a minimum, it will be important to see whether the same microbes can be found in other independently obtained plasma and serum samples from ALS patients and controls.

In summary, our analyses uncovered multiple sncRNAs whose abundance changes significantly between ALS patients and controls in both plasma and serum. Even if we focus solely on the sncRNAs that can be mapped to the human genome, we are still left with numerous sncRNAs that change statistically significantly between ALS patients and controls. These sncRNAs arise from well-annotated transcripts, namely, microRNA precursors, tRNAs, rRNAs, and Y RNAs, and and go beyond what has been explored in the ALS context. IsomiRs, tRFs, rRFs, and yRFs are ideal candidates for designing novel non-invasive biomarkers. Future efforts should include validation of these markers in larger and different cohorts of ALS patients and controls.

## Supplementary Information

Below is the link to the electronic supplementary material.

Supplementary file1 (XLSX 25 KB)

Supplementary file2 (XLSX 60 KB)

Supplementary file3 (XLSX 366 KB)

Supplementary file4 (XLSX 11811 KB)

Supplementary file5 (XLSX 253 KB)

Supplementary file6 (XLSX 4444 KB)

Supplementary file7 (XLSX 16 KB)

## Data Availability

(1) The primary datasets that were re-analyzed to generate the findings of this study are available through the Sequence Read Archive (SRA) of the National Institutes of Health (NIH). The datasets of the plasma collection, GEO project GSE168714 (Magen et al., 2021), are available at https://www.ncbi.nlm.nih.gov/Traces/study/?acc=PRJNA713778 and the datasets of the serum collection, GEO project GSE148097 (Dobrowolny et al., 2021), are available at https://www.ncbi.nlm.nih.gov/Traces/study/?acc=PRJNA623200 (2) The derivative datasets that were generated by this study are available in the supplementary material of this article in the form of separate Microsoft Excel files (Supp. Tables S1-7).
